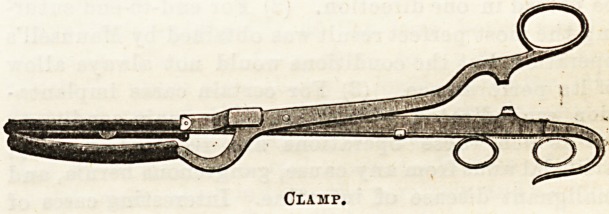# Progress in Surgery

**Published:** 1896-08-29

**Authors:** 


### Progress in Surgery.

### SURGERY OF THE INTESTINES.

**Wounds of the Intestine** are stated by Brown[1] to be comparatively small in cases of stab-wounds of the abdomen. Of 130 cases of this class in which the peritoneum was wounded less than one-third were complicated by wounds of the gut itself. Scudder[2] says that the best means of ascertaining the existence of penetration in these cases is by enlargement and careful investigation of the original wound. The use of the probe is misleading, and may give a false sense of security. A small parietal wound is not incompatible with grave intraperitoneal injuries, even though there be no shock. The possibility of intraperitoneal strangulation through the wounded omentum must be kept in mind. Interesting cases of recovery from severe wounds are reported by Woolsey[3] (sixteen perforations), Puzey[4] (penetration of gravid uterus), and Walker.[5]

**Intestinal Anastomosis and Resection.**—Several new instruments and "bobbins" are reported for aiding in lateral or end-to-end anastomosis of the intestine. Clarke[6] suggests a bobbin made with sloping ends, as in the accompanying figure. The pressure on the ends of the divided gut is made by means of india-

Clarke's Bobbin.

rubber rings. Both rings have the same diameter as the smaller part of the bobbin, *i.e.*, half an inch. They are cut so that when stretched to the diameter of the barrel of the empty bobbin their depth and width just brings them flush with the lips of the larger end of the bobbin. The mode of making the anastomosis is as follows: One of the rubber rings is lubricated and inserted into one end of the gut. and then a purse-string stitch is run close to the border of the intestine, and securely tied over the narrow neck of the barrel. The other end of the gut is treated in the same way, then the rings are easily pushed (by the fingers of the operator pressing through the gut) over the ends of the bobbin on to the barrel, and thus a secure anastomosis is effected. The redundant part of the mesentery may be drawn into two triangular folds, one on each side of the bowel, and fixed. The advantages of this method appear to be that it does away with the sharp cutting rim of Murphy's button, and having a larger lumen, is less likely to cause obstruction. Chaput[7] has also brought forward a new button, the advantage of which depend on the flexibility of the metal employed, which is pure tin. The general form of the instrument is that of an elliptical ring, pierced in its centre by an oval orifice. In profile it presents a circular groove, measuring about half an inch in width, and a third of an inch in depth. In the edge of this groove are three notches on each side, and the plates of metal between these notches are so thin that they can easily be bent so as to vary the distance between them, the width of the groove being thus regulated at the pleasure of the operator. In performing an entero-anastomosis, the author makes in each coil of the intestine a longitudinal incision of sufficient length for the admission of the button, and surrounds each incision by a purse-string suture which is drawn tightly round the circular groove, after the edges of the latter have been placed one within each intestinal coil. The lips of the groove are now pressed together by the fingers, applied to the outside of the intestine. This approximation of the lips of the groove gives the same result as a row of sutures, for the thin layers of tin, when brought together, cannot be separated without difficulty. As a further precaution, however, the author surrounds the button with sutures carried through the apposed serous coats at intervals of about half an inch. This button can also be used in cases of circular suture of the intestine. The author records three cases in which this instrument has been tried. Duplay and Cazin[8] have also suggested a method of anastomosis by means of two concentric metal cylinders, and they give diagrams illustrating the use of the apparatus, but at present the authors have not tried their instrument on man. Grant[9] has devised an ingenious clamp for facilitating lateral approximation by means of suture. The instrument consists of a clamp with five-inch blades, three-eighths of an inch in width, bearing between them a concealed knife, which can be withdrawn four inches. The blades are covered with rubber tubing to protect the bowel from injury by pressure.

A clasp at the end of the handles holds the blades compressed together as in the ordinary artery clamp. After a portion of gut has been excised, anastomosis is effected in the following manner: The two portions of gut are laid side by side, and the blades of the clamp are introduced into each portion of gut, so as to oppose when tightened the surfaces of the bowel opposite the mesentery, until the free ends of the bowel are received in the curve on the shank of the clamp. The clamp is tightened, and two interrupted stitches are inserted on each side near the middle of the blade, to prevent possible eversion of the edges of the gut after section; the knife is now made to traverse its route two or three times to insure complete section of the gut walls. While a second assistant holds the clamp, the surgeon, beginning about one inch from the free end, runs a glover suture rapidly along the upper surface, involving only the peritonea and muscular coats, and thus opposing the one-fourth inch peritoneal surfaces caught by the blades. By depressing the handles, the other surface presents at the resected point, and suture of the other side is completed in the same manner. The blades are now unlocked and withdrawn, and the finger explores to insure the patency of the opening. Invagination of the ends is now done in the usual manner, the cut surfaces of the mesentery sutured as may be necessary, and the anastomosis returned to the abdominal cavity. The advantages claimed for this method are that it makes the direct suture easy to any ordinary skilled hand, and does

Clamp.

away with any foreign body afterwards. Rogers[10] proposes a method of end to end anastomosis, which consists in turning back the peritoneal coat off one end of the small intestine, after resecting a portion of the gut, suturing the muscular coat thus exposed to the peritoneal coat of the other end of the intestine, subsequently turning down the reflected portion of peritoneum over the first row of sutures, which are thus completely buried, and suturing its deep fibrous surface to the outer serous surface of the unreflected end of the intestine. Thus a double sero-fibrous union is obtained which will unite both quickly and firmly.

The inner sutures are passed through the muscular coat of one end and the muscular and peritoneal coats of the other end of the bowel, which afford ample material for holding, whilst the outer sutures include the peritoneal coats only. The advantages claimed are (1) that it can be done with the aid of the instruments in a pocket case, and with very little assistance, and is therefore likely to be of special service in military surgery; (2) It is completed in only a little longer than the time required with the aid of such special appliances as bobbins; (3) The double sero-fibrous union is firm; (4) The mesenteric side is rendered safe from leakage. Robson,[11] speaking of twenty-six cases of enterectomy at the Leeds Infirmary, divided the series into three classes: (1) Those operated upon by simple suture, nine in number, of which five died, yielding a mortality of 55·5 per cent. (2) Those operated on by the Murphy button, five in number, of which one died, giving a rate of mortality of 20 per cent.; two had had fistula and a retarded convalescence; and in one the button had not passed while the patient was under observation. (3) Those in which a decalcified bone support in the shape of a button or some similar contrivance was employed to support the sutures, of which there were twelve cases with one death, giving a rate of mortality of 8·3 per cent. He said that before all other methods he preferred the use of a decalcified bone bobbin. The advantages, he claimed, were a saving of time from the use of only one or, at most, two sutures; the prevention of a subsequent stricture by the establishment of a continuous mucous canal; the perfect security against leakage; the absence of a foreign body in the intestine, as the bone dissolved when its work was done; the prevention of infection of the line of suture; the immediate continuity of the newly-made canal; and lastly, the applicability of the bobbin to any of the operations required for the establishment of continuity of the intestinal canal. Edmunds and Ballance[12] have made some experiments to determine the best method of uniting either bowel to bowel, or bowel to stomach. The conclusions arrived at were: (1) For lateral anastomosis Halsted's operation was best: a needle should be threaded at each end of each suture, the needles then had only to be passed in one direction. (2) For end-to-end suturing the most perfect result was obtained by Maunsell's operation, but the conditions would not always allow of its performance. (3) For certain cases implantation was indicated. There are three main conditions for which these operations are required—namely, artificial anus from any cause, gangrenous hernia, and malignant disease of intestine. Interesting cases of resection are recorded by McBurney,[13] Newman,[14] Durante[15] (for tuberculosis of the cæcum), and Allingham.[16]

**The Murphy Button.**—In considering the objections which have been urged against the contrivance, Murphy[17] maintains: (1) That the size of the opening made is not too small, since there is no tendency for it to contract; the No. 3 button will always suffice for operations on the small intestine, and the No. 4 will be called for only in dealing with the large intestine. (2) It is an impossibility for the button to give rise to obstruction unless some pathological contraction has pre-existed. (3) The great objection is that the lumen of the tube may become blocked. Of this accident the author knows only of three cases, and he considers that if the surgeon keeps the patient's bowels loose after the operation no such occurrence is likely to be met with. In conclusion, he points out that care must be exercised not to ligature too great an extent of the mesenteric vessels, otherwise sloughing and perforation are almost sure to follow. Everet[18] records a successful case of pylorectomy for fibroma, in which the intestine was afterwards joined to the stomach by means of Murphy's button.

**Perforating Typhoid Ulcers.**—Price[19] strongly advocates operation in these desperate cases. He records two successful cases which, with others he has collected, make a total of twenty-six cases operated upon, with eight recoveries. Hotchkiss[20] also speaks in favour of the operation and records a fatal case. Hollis[21] quotes Van Hook's conclusions: (1) When perforation occurs in typhoid fever the indication for operation is imperative; (2) the only contra-indication is a moribund condition of the patient; (3) collapse is often, at least temporarily, relievable by hot peritoneal flushing; (4) the stage of the fever is not to be considered as an indication, or as a contra-indication, for laparotomy; (5) the severity of the typhoid fever is alone not a contra-indication; (6) early laparotomy offers the most hope; (7) the symptoms of peritonitis should not be awaited before operating; (8) the pubished statistics of laparotomy for this condition are strongly in favour of operation.

**Mesenteric Cysts.**—Pagenstecher[22] reports two of these rare tumours. The first appears to have been a chyle cyst, although no examination of the wall was possible, on account of its intimate connection with the intestine, which compelled treatment by securing the cyst in the abdominal wound, and a secondary incision. The fluid had a fæcal odour from the absorption of fæcal gases. The second case is considered to be a cyst developed from a chronic inflammatory hyperplasia of a lymph gland, with hyaline degeneration, softening, and calcification, as was evident from the microscopical examination. Probably it had a tubercular origin. The cyst was as large as an apple, and lay in the mesentery, near the intestinal border, whence it was easily shelled out. These cysts are usually movable, and frequently mistaken for floating kidney. Echinococcus, serous, hæmorrhagic, chyle, dermoid, and these softened gland cysts are all found here.

[1] New York Med. Journ., Dec. 7, 1895. [2] Internat. Med. Mag., Nov., 1895. [3] Annals of Surg., April, 1896. [4] Lancet, April 26, 1896. [5] Annals of Surg., Jan., 1896. [6] Lancet, May 9, 1896. [7] Brit. Med. Journ., Jan. 18, 1896, and Sem. Med., No. 62, 1895. [8] Med. Chron., Jan., 1896. [9] Annals of Surg., Jan. 1896. [10] Brit. Med. Journ., April 11, 1896. [11] Ibidem, April 4, 1896, and Lancet, April 4, 1896. [12] Lancet, May 16, 1896, and Brit. Med. Journ., May 16, 1896. [13] Annals of Surg., April, 1896. [14] Glasgow Med. Journ., March, 1896. [15] Med. Rec., New York, March 7, 1896. [16] Med. Press and Circular, Jan. 29, 1896, and Feb. 5, 1896. [17] Practitioner, Feb., 1896. [18] New York Med Rec., March 14, 1896. [19] Therapeut. Gazette. May 15, 1896. [20] New York Med. Journ., Jan. 11, 1896. [21] Lancet, May 9, 1896. [22] Amer. Med. Surg. Bullet., March 14, 1896.

---

### DISEASES OF CHILDREN.

**The Treatment of Pneumonia.**—In broncho-pneumonia Dr. Gendre[1] considers it important that the air of the room should be frequently renewed and kept moist by means of steam from antiseptic fluids. In severe congestion of the lungs he advises local treatment by ice-bags rather than by vesication and hot applications. He has discontinued the use of anti-thermic drugs in pyrexia, having found quinine to be of no value, acetanilid dangerous, phenacetin inactive, and aconite too depressant, and relies on hydrotherapeutic measures. Amongst these he places great confidence in cold compresses employed in the following manner: Some layers of tarlatan or ordinary surgical gauze are dipped in water containing one-fourth part of alcohol, wrung out, and applied all round the thorax. Over this is placed a dry compress, and the whole is then fixed with a bandage or strapping. The water used is to be at the same temperature as that of the sick room, and the compress must therefore be changed frequently every quarter or half hour, as by that time it has got heated to the temperature of the skin. The cold bath is to be employed when the fever is excessive, the dyspnœa marked, and great nervous irritability present, but is to be avoided if a large part of the lung or lungs is involved or if the cardiac action is feeble or irregular. The temperature of the bath should not be above that of the ordinary living room. As cardiac stimulants he finds caffeine and alcohol give the best results, but notes that caffeine often produces cerebral excitement and insomnia. A series of articles on the treatment of pneumonia in children, based on the reports from six different children's hospitals in America, has recently been published[2], and will repay perusal. Speaking generally the treatment follows very similar lines to those pursued in this country. Expectorant medicines have been discarded for the most part, and antiseptic inhalations are used in place of them. Phenacetin has been found useful in relieving restlessness and procuring sleep. Pyrexia is treated by hydrotherapeutic measures, the cold bath, the cold pack, and cold sponging. Dr. Swift points out that it is important at the outset of cerebral symptoms to examine the ears carefully, as the exciting cause will often be found in an unsuspected otitis media.

**The Treatment of Hyperpyrexia by Apolysin.**—The depressing effect of antipyretics of the aniline series has led to Dr. Louis Fischer[3] to dispense with them entirely and trust to hydrotherapeutics; but he is now of opinion that in apolysin he has found a reliable antipyretic which has no injurious effect on the system. This drug was introduced by Dr. F. von Heyden[4], and is a yellowish white crystalline powder of a slightly sour taste, sparingly soluble in water (1 in 55), but very soluble in glycerine and in alcohol. Dr. Fischer has now employed apolysin in thirty-eight cases, and his description of eight of these, comprising cases of pneumonia, typhoid fever, rheumatic fever, and measles, tends strongly to support his views as to the value of the drug. He did not confine his investigation solely to the condition of hyperpyrexia, which in children is a somewhat elastic term, but found that in ordinary pyrexia also apolysin acted beneficially. The following is his rule for the dosage: for a child of one year five grains of apolysin, to be repeated every two or three hours, the intervals depending on the urgency for the reduction of the hyperpyrexia. If there is no distinct effect from the five-grain dose after three doses have been given, it is perfectly safe to give ten grains every two hours until the fever has been reduced. When the drug is administered per rectum the dose should be doubled. He has had no experience of any bad effects such as cardiac weakness, fall of the temperature below normal, gastric disturbance, or a tendency to a cumulative effect even after large doses. He advises that the drug be pushed until the temperature begins to fall or diaphoresis sets in.

**Syphilis.**—In the Hunterian lectures on "Infantile Syphilis," Dr. J. A. Coutts[5] discusses some of the problems connected with the hereditary nature of this affection. In considering the manifestations of the disease he lays stress on wasting from the time of birth as frequently the earliest sign of syphilis. This wasting is of grave importance, because it is dependent on atrophy of the mucous and muscular coats of the stomach and intestine, and unless it is treated early the hope of cure is small. In the case of children who are emaciated at birth the prognosis is almost always hopeless, but if a well-nourished infant commences to waste soon after birth, then, in the absence of any recognisable cause, and even in the absence of any other sign of syphilis, Dr. Coutts thinks that anti-syphilitic treatment should be employed. Swelling of the nasal mucous membrane with "snuffles" is usually the first objective sign in infantile syphilis, but as a similar condition is frequently due to some transient cause, too much weight must not be placed on this sign. The skin eruptions are of great variety and importance. One of the rarer forms is that known as the "syphilitic carbuncle," which is often the sole manifestation of the later stages of inherited syphilis. It consists of an elevation of the epidermis of the size of half a hazel nut, purple in colour, and situated usually on the outer and upper aspect of the thigh. He has not found enlargement of the spleen present to an excessive degree in syphilis, and believes it is never so marked as to lead to changes in the blood. When these changes are present in a syphilitic child, he believes that rickets is also usually present, and is the prime factor. As regards the skull, the "natiform" condition, with four bosses closely surrounding the anterior fontanelle, is diagnostic of syphilis, while an exaggeration of the frontal and parietal eminences is usually rhachitic. After the age of six months a combination of these two diseases is frequently present, and the form of the skull is modified by both of them in a manner which makes the differentiation of the etiological factors very difficult.

Syphilitic epiphysitis of the long bones usually occurs in infants of from three to six months, and affects the upper more than the lower extremity. He does not regard this epiphysitis as the invariable cause of "syphilitic pseudo-paralysis," and suggests that there may be a real nervous affection of peripheral origin which induces the paralysis, and also the wasting of the muscles, which is often a prominent feature. There are many other controversial points discussed in these lectures, which space prevents our touching on, but which are well worthy of study by those interested in the subject. Of 148 children suffering from congenital syphilis, and examined during the first year of life, Dr. Hochsinger[6] found a decided enlargement of the liver in forty-six. Of these forty-six hepatic cases, thirty recovered under anti-syphilitic treatment, and sixteen died, or were lost sight of. In one fatal case he found also tuberculous caseation in the liver, and he notes that this is the first recorded case of a child having both congenital syphilis and congenital tuberculosis. On clinical examination the liver was usually smooth and hard, jaundice and ascites were absent, the spleen was enlarged as a rule, and the accompanying manifestations of syphilis were seen in the skin, the nose, and the bones. Treatment, in most of the cases, led to a reduction in the size of the liver, and less rapidly of the spleen also. The pathological changes observed were a diffuse affection of the hepatic parenchyma, consisting of inflammatory cells amongst the individual liver cells, and some thickening of the intima of the arteries. In an interesting paper, Dr. B. Sachs[7] discusses "the nervous manifestations of hereditary syphilis in early life." These are apt to be associated with a cerebro-spinal lesion, extensive in its distribution, but of no great intensity, and characterised by remissions and relapses. The underlying morbid changes are a specific thickening of the pia mater in any part of its course, and a specific endarteritis with consequent softening or hæmorrhage. The most common clinical type is marked by spastic paraplegia, which occurs early in life without any special assignable cause is subject to relapses and recoveries, and is often associated with cerebral symptoms, such as local palsies or complete immobility of the pupils.

**Retropharyngeal Abscess.**—Dr. Henry Koplik[8] communicated a paper on this subject based on an experience of 76 cases, to the New York Academy of Medicine. The source of the affection is frequently to be traced to some lesion of the tonsils, the mouth, or the naso-pharynx. The condition is most common during the period of suckling, and is rare after the second year. The signs are alteration in the cry, refusal of food, stiffness of the neck, difficulty in breathing, and, on digital examination, a swelling in the throat. Many cases did well if left to burst, as the opening was usually small at first, and the pus escaped so gradually that the risk of suffocation was avoided. He had treated some cases by opening the abscess internally, the patient's head being directed downwards so as to allow the pus to escape from the mouth. An external incision was to be preferred when the deep cervical glands were affected and there was a large swelling in the neck.

**Night Terrors (Pavor Nocturnus).**—This subject is discussed by Dr. J. A. Coutts,[12] who considers that a distinction should be made between nightmare and night terrors. Night terrors are dependent on a central cerebral disturbance (idiopathic), and occurs in children between the ages of two and eight years more especially in those with a neurotic family history. There is often a preceding history of infantile convulsions, and a subsequent history of epilepsy. In an attack the patient starts screaming in great terror, "sees visions," does not awake, and has no recollection of the occurrence on the following morning. In nightmare, on the other hand, the cause is usually to be found is some reflex disturbance from the stomach, throat, &c., and in an attack the child wakes dreaming dreams but not seeing visions, can be soothed to rest again, and recollects the occurrence next morning. There is no limit as to age, and no evidence of nervous disease in the patient or family. The treatment in nightmare will therefore be to remove the cause, and in night terrors to regulate the nervous system by bromides, &c.

[1] L'Abeille Medicale, March 14, 1896, and Therap. Gazette, June 15th, 1896. [2] Arch. of Pediat, April, 1896. [3] N. Y. Med. Rec., Feb. 22, 1896, [4] Allgem. Medic., Central Zeitung, Nos. 60-62, 1895. [5] The Lancet, April 11, 18, and 25, 1896. [6] Wien. Med. Wochen, 1896, Nos. 9 to 14, and Ep. B. M. J., May 9, 1896. [7] Amer. Med. Surg. Bulletin, Feb. 15, 1896. [8] Pediatrico I., 1896, p. 461. [9] Am. Jour. Med. Sci., Feb., 1896.

## Figures and Tables

**Figure f1:**
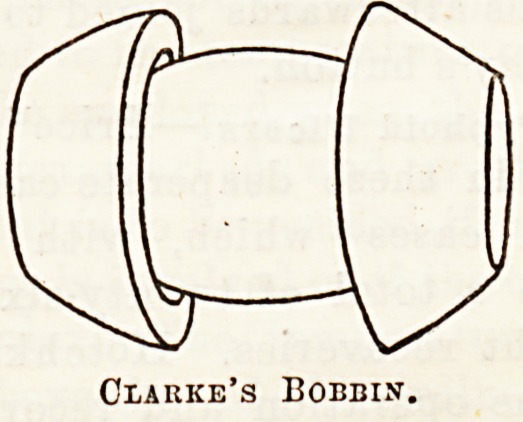


**Figure f2:**